# SAMD9/9L突变致儿童全血细胞减少性疾病5例报告及文献复习

**DOI:** 10.3760/cma.j.issn.0253-2727.2023.04.014

**Published:** 2023-04

**Authors:** 倩雯 尚, 莲 薛, 乐萍 张, 爱东 陆

**Affiliations:** 北京大学人民医院儿科，北京 100044 Pediatrics Department Peking University People's Hospital, Beijing 100044, China

SAMD9与其旁系同源基因SAMD9L并排位于染色体7q21上，功能上有58％的相似性[1]，均表达IFN和TNF-α，具有生长抑制因子的功效[2]。SAMD9/9L突变与血细胞减少、骨髓衰竭、骨髓增生异常综合征（MDS）及免疫缺陷相关[3]–[4]，遗传学检查上可表现伴或不伴有7号染色体单体或7染色体部分单体缺失（长臂缺失）（−7/7q−）的SAMD9/9L的错义突变或截断突变。SAMD9/9L在全血细胞减少中突变发生率为18.6％[5]，在MDS中突变发生率为8％～17％[6]–[7]。SAMD9/9L突变可通过等位基因丢失、回复突变或单亲二倍体（UPD）复制等对骨髓造血功能进行修正[8]–[9]。SAMD9/9L突变所致疾病临床表现异质性强，目前治疗标准不统一，欧洲儿童骨髓异常增生综合征工作组（EWOG-MDS）建议，没有严重血细胞缺乏和不依赖输血的SAMD9/9L突变患者，可采用观察及等待策略；进展为髓系恶性肿瘤者及时行造血干细胞移植（HSCT）结果令人满意[10]。本研究中，我们对我院儿科SAMD9/9L突变致儿童全血细胞减少5例患者的临床表现、实验室检查、治疗及转归进行描述。

## 病例与方法

1. 病例资料：回顾性分析2019年1月至2022年1月我院儿科以血细胞减少起病且SAMD9/9L突变的患儿资料。全血细胞减少定义为中性粒细胞绝对计数（ANC）<1.50×10^9^/L、PLT<100×10^9^/L且HGB<同龄儿的2个标准差。本研究获得我院伦理委员会批准（批件号：2023PHB084-001），患儿的检查及治疗均已取得家长的知情同意。

2. 实验室检查：抽取患者外周血送检全血细胞计数；患儿父母及患儿外周血或骨髓液送检全外显子基因检测及家系验证；骨髓穿刺送检骨髓涂片、免疫分型、分子学诊断及染色体G显带法或FISH检测，同时送检骨髓活检进行病理学诊断。

3. 疗效评估：（1）急性白血病：完全缓解（CR）定义为骨髓原始幼稚淋巴细胞<5％，ANC>1.50×10^9^/L且PLT>100×10^9^/L），外周血无原始幼稚淋巴细胞且无髓外白血病证据；复发定义为获得CR后再次出现骨髓原始幼稚淋巴细胞>5％，或出现髓外白血病。（2）再生障碍性贫血（AA）：疗效判定参照儿童获得性AA诊疗建议[11]。CR：脱离血制品输注条件下，HGB>110 g/L、ANC>1.50×10^9^/L且PLT>100×10^9^/L；部分缓解（PR）：脱离血制品输注，HGB>80 g/L、ANC>0.50×10^9^/L且PLT>20×10^9^/L；无缓解（NR）：未达到PR、CR标准。（3）MDS：根据MDS国际工作组（IWG）疗效标准[12]，评估MDS的自然病程、细胞遗传学反应、血液学改善和生存质量改善。

4. 随访：对患儿定期门诊及电话随访，每3个月随访1次，随访内容包括疗效、有无复发、疾病转归、死亡情况、死亡原因等。随访截止时间为2022年4月13日。

## 结果

1. 一般资料及临床表现：5例SAMD9/9L突变患者中男2例、女3例，中位年龄2岁（1岁2个月～10岁），起病至确诊时间5 d～2年，中位随访11（3～37）个月。疾病类型：MDS 2例，AA 1例，先天性骨髓衰竭（IBMF）1例，急性B淋巴细胞白血病（B-ALL）1例。反复感染（3例）是最常见伴随症状，部分患者存在生长发育异常（2例）及神经系统症状（2例），所有患者均无外生殖器异常（表1）。

**表1:** 5例伴SADM9/9L基因突变血细胞减少患儿的一般情况、临床特征、治疗及转归

例号	性别	诊断年龄	起病至诊断时间	诊断	起病时三系减低	反复感染	发育异常及神经系统症状	外生殖器异常	其他	治疗方案	是否移植	转归
1	女	2岁	2年	MDS-EB1	+	+	–	–	–	阿糖胞苷+阿扎胞苷+地塞米松、D-CAG方案	allo-HSCT	随访11个月无病存活
2	男	1岁2个月	1年	MDS-U	+	+	–	–	–	观察，IVIG	allo-HSCT	随访37个月无病存活
3	男	10岁	5 d	VSAA	+	–	–	–	肝炎、过敏性鼻炎、过敏性哮喘	甲泼尼龙+环孢素A+艾曲泊帕	全相合非血缘HSCT	随访8个月无病存活
4	女	8岁	1周	B-ALL	+	–	–	–	反复皮肤过敏	VDLD方案诱导，甲氨蝶呤、阿糖胞苷等巩固	否	随访12个月无病存活
5	女	2岁	2年	IBMF	+	+	+	–	–	观察	否	随访3个月存活

**注** MDS-EB1：骨髓增生异常综合征伴原始细胞增多1；MDS-U：骨髓增生异常综合征不能分类；VSAA：极重型再生障碍性贫血；B-ALL：急性B淋巴细胞白血病；IBMF：先天性骨髓衰竭；D-CAG方案：地西他滨、阿糖胞苷、阿克拉霉素、G-CSF；IVIG：静脉注射免疫球蛋白；VDLD方案：长春新碱、地塞米松、去甲氧柔红霉素、左旋门冬酰胺酶；allo-HSCT：异基因造血干细胞移植

2. 实验室检查：所有病例骨髓涂片可见巨核系减少，3例未见原始细胞，骨髓增生活跃，但可见增生不良表型，2例可见原始细胞，分别占9％、94％。免疫分型均未见阵发性睡眠性血红蛋白尿（PNH）克隆。免疫球蛋白、补体、淋巴细胞亚群均未见明显异常。1例患者有肝炎病史，但自身免疫性肝炎及狼疮抗体检查未见异常。5例中检测到SAMD9/9L突变基因共6个，其中SAMD9L突变4个，SAMD9突变2个。根据其测序深度、突变类型及突变来源，其中SAMD9L胚系突变3个，体细胞突变1个；SAMD9均为胚系突变。美国医学遗传学会（ACMG）标准，疑似致病变异3个，临床意义未明变异（VUS）3个。伴有7号染色体单体（−7）1例，检测出有伴随基因3例（表2）。

**表2:** 5例全血细胞减少患儿SAMD9/9L突变信息及实验室检查资料

例号	骨髓涂片	免疫分型	伴随基因	染色体核型	骨髓活检	突变基因	变异来源	纯合/杂合	变异类型	氨基酸改变
1	原始细胞占9%	无PNH克隆	RUNX1、WT1、EVI1、PRAME	45,XX,−7[20]	MDS可能	SAMD9L	自发	嵌合	胚系	p.K1532N
						SAMD9L	自发	杂合	体系	p.E685del
2	未见原始细胞	无PNH克隆	SPTB、FANCG	46,XY[20]	造血组织增生活跃	SAMD9	母亲	杂合	胚系	p.N720Tfs*34
3	增生不良，巨核系减少	无PNH克隆	未检出	46,XY[4]	造血组织增生低下，巨核细胞少	SAMD9L	母亲	杂合	胚系	p.A189V
4	原始幼稚淋巴细胞占94%	幼稚B细胞占4.61%，无PNH克隆	IgH、IKZF1、CSRP2	46,XX[20]	未进行活检	SAMD9L	母亲	杂合	胚系	p.T1254I
5	红系增生低下，巨核系减少	无PNH克隆	未检出	46,XX[20]	组织造血红系、巨核系增生低下	SAMD9	自发	杂合	胚系	p.Q1198H

**注** PNH：阵发性睡眠性血红蛋白尿症；MDS：骨髓增生异常综合征

3. 治疗和转归：3例患者行HSCT治疗，包括MDS（2例），AA（极重型）（1例），确诊至移植时间为3～20个月，其中异基因HSCT（allo-HSCT）2例，全相合非血缘性HSCT 1例；3例移植后随访血常规、骨髓穿刺均提示CR，至末次随访均处于无病存活状态（分别随访11、37、8个月）。确诊B-ALL 1例，VDLD方案诱导化疗，巩固强化方案包括大剂量甲氨蝶呤、大剂量阿糖胞苷等序贯交替使用，规律复查骨穿均提示CR。确诊IBMF 1例，采用观察及随访策略，定期复查血常规示血小板逐渐上升，至末次随访PLT为95×10^9^/L。

## 讨论

1978年Li等[13]在ATXPC综合征中发现SAMD9L突变，2016年Narumi等[14]在MIRAGE综合征中发现SAMD9突变。SAMD9/9L具有生长抑制因子功效，可被Ⅰ型和Ⅱ型干扰素上调[15]，发挥抗病毒及抑制感染的功能，SAMD9/9L突变多为功能获得性突变[9]，抑制组织细胞增长，可出现反复感染、骨髓细胞增殖下降及生长发育迟缓等[16]。作为骨髓衰竭中常见基因，SAMD9/9L突变与GATA2突变发生率相似，分别为8％和7％[17]，但不同的是，SAMD9/9L突变往往在生命早期出现难治性血细胞减少症或骨髓造抑制。近年来在一些伴有−7/7q−的MDS中发现SAMD9/9L突变[7]，之后有关报道开始不断出现，表3汇总了2017至2021年间SAMD9/9L突变研究报道[5]–[6],[17]–[25]。目前为止最大宗的儿童数据来自EWOG-MDS协作组[17]，669例MDS患者中67例（10％）携带胚系SAMD9/9L突变。其中一组548例MDS患儿的队列中检测出SAMD9/9L突变42例，占该队列中−7/7q−总数的38％，其中SAMD9突变26例，5年生存率为84％，SAMD9L突变16例，5年生存率93％。根据ACMG标准，该研究中VUS最多（72％），致病性变异最少（9％）。HSCT治疗效果好，总生存率为85％。MIRAGE综合征较罕见（2例），HSCT后均死亡；免疫抑制治疗（2例）及观察随访（13例）生存结局良好。另外该研究发现伴SAMD9/9L突变的MDS和其他MDS亚组之间的HSCT总生存率相似。

**表3:** 2017—2021年关于伴有SAMD9/9L突变综合征或非综合征相关疾病临床数据报道

文献来源	发表时间	地区	诊断	治疗方案	转归
Buonocore等[18]	2017	英国	MIRAGE综合征（8例）	2例接受移植，6例等待与观察	2例移植均存活，6例死亡
Schwartz等[19]	2017	美国	MDS（4例）	未提及	未提及
Sangita等[6]	2018	德国	MDS（43例）	43例接受移植	38例存活，5例死亡
Nagata等[20]	2018	日本	MDS（21例）、BMF（3例）	未提及	未提及
Pastor等[21]	2018	德国	MDS（7例）	5例接受移植，2例等待与观察	6例存活，1例移植后死亡
Bluteau等[5]	2018	法国	BMF（16例）	10例接受移植，5例等待与观察，1例失访	13例存活，其中6例自发缓解，12例移植后死亡，例失访
Wong等[22]	2018	美国	MDS（1例）、AML（2例）、Transient Mo7（2例）	1例接受移植，2例化疗，2例等待与观察	3例存活，其中2例自发缓解，1例化疗存活，1例移植后死亡，1例化疗后死亡
Sarthy等[23]	2018	美国	MIRAGE综合征伴BMF（1例）、伴MDS（5例）	6例接受移植	3例死亡，3例存活
Sahoo等[17]	2021	欧洲	MDS（67例）	52例接受移植，2例IST，13例等待与观察	59例存活，8例非复发死亡
Ahmed等[24]	2019	美国	MDS（10例）、CAMT（1例）、DCK（1例）	12例接受移植	10例存活，1例移植后复发死亡，1例移植后并发症死亡
Yoshida等[25]	2020	日本	JMML（1例）、MDS（2例）、中性粒细胞减少症（1例）	4例接受移植	2例存活，1例死亡，1例失访

**注** MIRAGE综合征：包括骨髓增生异常、反复感染、生长受限、肾上腺发育不全、生殖器异常和肠病；MDS：骨髓增生异常综合征；BMF：骨髓衰竭性疾病；AML：急性髓系白血病；Transient Mo7：暂时性7号染色体染色单体；CAMT：先天性无巨核细胞血小板减少症；DCK：先天性角化不良；JMML：幼年型粒单核细胞白血病

近年来有研究发现伴SAMD9/9L突变全血细胞减少的患者在病程演变中出现了自发缓解，关于该发现目前有两种机制解释：首先，因7号染色体长臂的单亲二倍体（UPD7q）形成，野生型等位基因替换突变位点恢复骨髓造血功能；另外，顺式体细胞功能丧失性突变或回复突变使突变基因丢失恢复骨髓造血功能[26]。疾病治疗方面，因SAMD9/9L突变临床表现差异大、治疗方式各不相同，目前并无前瞻性管理指南，根据EWOG-MDS工作组推荐，对于临床症状轻且无严重中性粒细胞减少或输血依赖的难治性血细胞减少患者，可采用观察和等待策略，定期检测血常规，每年1次骨髓评估检测高危体细胞变化（如−7或体细胞驱动突变）[10]；伴有−7/7q−非综合征患者及时行HSCT移植结局较良好[23]，但综合征患者治疗需慎重，MIRAGE综合征背景下接受HSCT的患者生存率低[27]；ATXPC患者HSCT后仍会进行性出现神经系统症状且预后不良[9]。MDS患者HSCT后的5年生存率可达85％[28]。本文中的5例非综合征患者，临床表现差异大，1例临床症状较轻的患者目前采用观察和等待策略，定期复查血常规出现血小板逐渐回升；3例HSCT治疗效果较好，目前存活良好。5例患者病程中体现了SAMD9/9L突变的特点，1例患者与其同卵双胎姐姐具有相同SAMD9L（p.K1532N）胚系突变位点，患儿及其双胎姐姐均反复感染，其姐因重症感染死亡，该患儿行HSCT治疗后存活良好，检测发现患儿体内存在另一个SAMD9L（p.E685del）体细胞突变位点，我们猜测该突变可能为功能缺失性突变，部分修正了胚系功能获得性突变，改善了患儿的生存结局。另外，目前报道SAMD9/9L突变在骨髓恶性肿瘤中常见，淋系恶性肿瘤报道罕见，国外一篇文献报道在B-ALL先证者[29]的淋巴母细胞系中发现了功能获得性的SAMD9L突变，经荧光原位杂交（FISH）测出肿瘤易感融合基因ETV6-RUNX1，功能验证后发现B-ALL是SAMD9L突变与ETV6-RUNX1融合基因共同作用的结果。本组B-ALL患儿也检测出IKZF1等淋系白血病相关突变，我们推断该患儿的B-ALL疾病的发生可能是SAMD9L与其他融合基因共同作用的结果。
